# Kinematic and Kinetic Patterns Related to Free-Walking in Parkinson’s Disease

**DOI:** 10.3390/s18124224

**Published:** 2018-12-01

**Authors:** Martín Martínez, Federico Villagra, Juan Manuel Castellote, María A. Pastor

**Affiliations:** 1 Neuroimaging Laboratory, Division of Neurosciences, CIMA University of Navarra, Applied Medical Research, 31008 Pamplona, Spain; mmvillar@unav.es (M.M.); fev1@aber.ac.uk (F.V.); 2 School of Education and Psychology, University of Navarra, 31008 Pamplona, Spain; 3 Ergonomics and Human Factors Unit, National School of Occupational Medicine, Carlos III Institute of Health, 28029 Madrid, Spain; jmcastel@isciii.es

**Keywords:** Parkinson’s disease, free-walking, bilateral coordination, kinematics, kinetics, pressure-sensitive insole sensors, machine learning

## Abstract

The aim of this study is to compare the properties of free-walking at a natural pace between mild Parkinson’s disease (PD) patients during the ON-clinical status and two control groups. In-shoe pressure-sensitive insoles were used to quantify the temporal and force characteristics of a 5-min free-walking in 11 PD patients, in 16 young healthy controls, and in 12 age-matched healthy controls. Inferential statistics analyses were performed on the kinematic and kinetic parameters to compare groups’ performances, whereas feature selection analyses and automatic classification were used to identify the signature of parkinsonian gait and to assess the performance of group classification, respectively. Compared to healthy subjects, the PD patients’ gait pattern presented significant differences in kinematic parameters associated with bilateral coordination but not in kinetics. Specifically, patients showed an increased variability in double support time, greater gait asymmetry and phase deviation, and also poorer phase coordination. Feature selection analyses based on the ReliefF algorithm on the differential parameters in PD patients revealed an effect of the clinical status, especially true in double support time variability and gait asymmetry. Automatic classification of PD patients, young and senior subjects confirmed that kinematic predictors produced a slightly better classification performance than kinetic predictors. Overall, classification accuracy of groups with a linear discriminant model which included the whole set of features (i.e., demographics and parameters extracted from the sensors) was 64.1%.

## 1. Introduction

Gait impairment represents a classical hallmark of Parkinson’s disease (PD) in the early stages of the illness [1,2,3] and over time becomes one of the most incapacitating signs [4]. Different gait features are affected, such as force control [5,6,7,8], load sensitivity [9], and interval timing [10], and worsen with disease progression. In the specific case of PD, an appropriate assessment of gait is a requirement in order to evaluate function and may also direct medical interventions, being of special interest from mild PD stages during the ON-clinical status. 

The quantification of bilateral coordination on gait has been generally measured through locomotion tasks under non-natural conditions and with the help of instruments such as driving belts [11,12] or pedaling devices [13]. Such studies have demonstrated that healthy subjects are able to control each leg separately while this ability declines in PD [14]. 

Most studies on gait in PD have been undertaken to quantify relevant kinematic characteristics of movement such as asymmetry and the phase coordination [15], whereas the dynamics underlying ground reaction forces (GRFs) have not been fully addressed [14]. This would open an opportunity to establish the relationships between kinetics and kinematics in order to evaluate the altered gait pattern in PD. During the last decade, several studies have implemented machine learning approaches in order to distinguish free-walking between healthy subjects and PD patients by recording trunk acceleration [16], GRFs [17,18,19,20,21,22,23], or by using alternative systems such as smartphone based [24] or wearable inertial devices [25]. Often, literature on gait in PD does not specify their clinical characteristics: duration of the disease, clinical impairment scores, and whether they were explored under the effect of dopaminergic medication. Consequently, the interactions between the kinematics and the kinetics of free-walking in PD patients under the effect of levodopa therapy are not fully explored. Considering the uncomplicated use and their comfort, in-shoe pressure wireless measurement systems are one of most promising tools for quantifying the GRFs related to locomotion in real-world settings [26]. 

Accordingly, the objective of the present paper is to compare the kinematics and kinetics of free-walking at a natural pace between mild PD patients, during the clinical ON status, and two control groups, corresponding to healthy young and age-matched healthy volunteers with the use of in-shoe pressure sensors. Moreover, we assess the capabilities of the kinematic and kinetic features extracted from the plantar sensors to classify individuals from each group by means of machine learning approaches. We hypothesize that gait parameters would differ across these groups and that such differences will help us to understand the gait characteristics in PD patients during the clinical ON status.

## 2. Materials and Methods

### 2.1. Subjects

A total of thirty-nine individuals, divided into three groups participated in this study: sixteen healthy young control (YC) subjects (seven female), eleven medicated Parkinson’s disease (PD) subjects (disease duration 4 ± 1.8 years; one female) with no history of falls and twelve age-matched control (AMC) subjects (four female). After providing informed consent and prior to the completion of the task, participants were examined by a neurologist. Both YC and AMC had a normal vision and absence of any neurological, psychological or motor disorder. PD patients did not present signs of abnormal postural reflexes or falls. All subjects were right-handed as assessed by the Oldfield Handedness inventory [27]. The recordings were made in the University of Navarra, School of Medicine Hospital, Clínica Universidad de Navarra. Table 1 shows the demographic characteristics of the groups.

In the PD patients’ group, the Unified Parkinson Disease Rating Scale (UPDRS -motor Section III) median score was 15 ± 4.28. Four patients were predominantly left hemi-body affected (clinical onset, AFF) and all patients had stable response, lasting more than three hours to medication. The levodopa equivalent daily dose (LEDD) in this group was 400 ± 327 mg, and the Mini-Mental State Examination (MMSE, [28]) median score was 29 ± 1.63. The protocol was approved by the Ethics Research Committee of the University of Navarra.

### 2.2. Experimental Procedure

Participants were requested to walk over a 25-m walkway at their preferred cadence during 5 min while pressure beneath each foot was recorded. At both ends of the walkway, two marks were placed on the ground to indicate the participants on the corresponding turn. An individual pace was chosen because at least in healthy subjects, variability is minimized at this rate [29]. Spatial measures (i.e., average velocity and step length) were estimated from the total displacement divided by task duration and number of steps, respectively.

### 2.3. Data Acquisition

Pressure data on both plantar surfaces were collected using the F-scan in-shoe pressure measurement system (Tekscan, Boston, MA, USA) that included 0.15 mm flexible insoles with a resolution of four sensors per square centimeter and a total of 954 sensors per insole. Plantar pressures under each foot were used to derive kinematic and kinetic parameters offline. Prior to registration, insoles were calibrated and adapted individually to the shoes of each participant, which used the same type of sneakers for the task in order to minimize differences in footwear. During recordings, data from both insoles were synchronized and instantly sent via wireless to a laptop computer, which registered them with a sampling frequency of 100 Hz.

### 2.4. Data Analysis

Spatio-temporal and GRF gait parameters were extracted and analyzed with Matlab 2018a (The MathWorks, Inc., Natick, MA, USA). For each individual, the time series corresponding to the vertical component of the GRF was extracted by summing the data from all the sensors of the corresponding insole. To smooth out spurious long-term trends, data were filtered with a simple moving average filter (window of 5-samples). Then, we calculated the initial and final temporal contacts of each limb with the ground by determining the local minima corresponding respectively to foot-strike and toe-off of each stride cycle. An automated median filtering procedure was applied to eliminate outliers with respect to the mean of each time series in order to study the intrinsic dynamics of free-walking. The outliers were typically steps that occurred at the end of the hallway (i.e., turns and contiguous strides).

#### 2.4.1. Kinematics

The following parameters were calculated for the right and left GRF time series: stride time (time from initial contact—typically heel strike—to the next initial contact of the same foot with the ground), stance time (time from initial contact of one foot with the ground to its final contact), swing time (time that one foot is on the air), percentage of stance time (PST, 100 × stance time/stride time) and percentage of swing time (PSWT, 100 × swing time/stride time). Moreover, double support time (DST) was determined as the time of bilateral foot contact. Stride-to-stride variability values were determined from the coefficient of variation (CV; CV = 100 × standard deviation/mean) of the stride, stance, and swing times and also DST. Gait asymmetry (GA) was evaluated by comparing the swing times performed by each leg according to the following expression:

(1) $$GA=100\times \left|\ln\left(\frac{SSWT}{LSWT}\right)\right|$$

where *SSWT* and *LSWT* represent respectively the mean swing time for the leg with the short and the leg with the long swing time [14].

Bilateral coordination was determined by quantifying the phase between the step timing of the left and right legs (ideally 180°, [13]). The phase of *i*-th cycle [*φ_i_*, in degrees] was determined as the normalized step time with respect to the stride time of *i*-th cycle. The leg with the higher value of the average swing time was used as reference in order to preserve uniformity across subjects. Thus, *φ_i_* was defined as:

(2) $$\phi_{i}=360\times \left|\frac{t_{Si}-t_{Li}}{t_{L(i+1)}-t_{Li}}\right|$$

where *t_Si_* and *t_Li_* represent the time of the *i*-th heel strike of the leg with the short and the leg with the long stride time, respectively, and *t_L_*_(*i*+1)_ > *t_Li_* > *t_Si_*. In order to characterize both accuracy and consistency in phase generation, the phase coordination index (PCI) was defined as the sum of coefficient of variation of *φ_i_* and the mean absolute difference between *φ_i_* and 180° (i.e., *φ* deviation). Finally, individual’s cadence was estimated through the periodogram—a method to estimate the spectral density of a signal—and calculated as the dominant frequency of the power spectral density of the GRF time series. Kinematic parameters were averaged across the corresponding individuals to get representative group values. A total of 28 parameters composed kinematic data.

#### 2.4.2. Kinetics

Valid gait cycles were extracted from the right and left foot GRF time series and individually segmented using the contact times determined in kinematic analyses. Then, individual cycles were averaged and normalized by dividing by the weight of participants. In order to obtain a representative GRF pattern for each group, cycle patterns were averaged across individuals. Considering the M-shape of GRFs within a gait cycle; three force and three time parameters were used to characterize group patterns. Heel-peak force (HPF) was defined as the first peak after the foot touches the ground, corresponding to the force when the subject transfers its entire body weight on one leg. Toe-off force (TOF) was defined as the last peak before toe-off, i.e., the peak during forefoot contact. Mid-stance force (MSF) was defined as the lowest force between HPF and TOF. Similarly, the time to heel-peak force (HPT) was defined as the time from initial contact to the time of HPF. The time to toe-off force (TOT) was defined as the time from the initial contact to the time of TOF. The time to mid-stance force (MST) was defined as the time from initial contact to the time of MSF. Those temporal measures are presented as percentages of the stance cycle. The coefficients of variation of kinetic parameters were also computed. A total of 24 parameters composed kinetic data.

### 2.5. Statistical Data Analysis

All statistical data analyses were performed in Matlab 2018a. To examine statistical differences between groups on gait, Kruskal-Wallis tests [30] were applied on the demographic, kinematic and kinetic parameters. If any test showed significant group differences (*p* < 0.05), Wilcoxon rank sum tests were performed to compare two groups at a time. 

In order to provide a better understanding of the underlying disturbed processes to parkinsonian gait, feature selection analyses were performed on the parameters highlighting in PD patients separately for each group while including the demographic, kinematic and kinetic parameters as predictors for all groups, and also including the clinical status in PD patients. We used the ReliefF algorithm [31] as it allows finding the minimally sized feature subset that is necessary and sufficient to describe a target concept [32]. This algorithm allows detecting statistically relevant features in noisy data, being notably sensitive to feature interactions [32,33]. A value of k = 10 was selected because it has been adopted as the default setting based on preliminary empirical testing [33].

Automatic classification based on machine learning was performed in the Classification Learner Toolbox with the aim of assessing the type of data that better recognize gait changes due to ageing and PD and also to evaluate the capability of the GRF system to predict ageing and PD. Thus, group was modeled as response variable and four classification datasets were included as predictor in different analyses: (I) kinematics (i.e., 28 parameters), (II) kinetics (i.e., 24 parameters), (III) kinetics and kinematics (i.e., 52 parameters), and (IV) demographics + kinematics + kinetics (i.e., 56 parameters). Datasets with combined features were classified followed by implementation of data fusion. To improve the accuracy in group classification, a linear discriminant analysis (LDA, [34]) was used, as this method resulted on a higher accuracy in comparison to the other predictive models (see Appendix A and Supplementary Table S1). A 39-fold cross validation (i.e., leave-one out) scheme was employed for all the analyses.

## 3. Results

Anthropometric characteristics of the study groups are presented in Table 2. Patients and elder controls were significantly older than young controls. Population sample were predominantly male. Young controls, elders and patients did not significantly differ with respect to height, step length, speed or cadence; however, on average, patients had a tendency to a higher weight and body mass index than young control subjects. 

### 3.1. Kinematics

**Figure 1:**
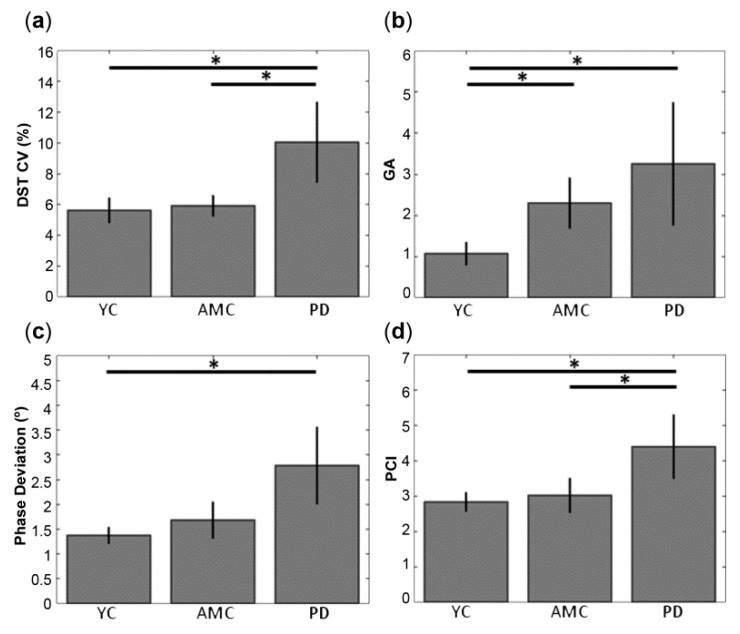
Kinematic parameters showing significant statistical differences between groups during free-walking: (**a**) Double support time coefficient of variation (DST CV), (**b**) Gait asymmetry (GA), (**c**) *φ* deviation and (**d**) phase coordination index (PCI). * *p* < 0.05.

Spatio-temporal features related to gait for YC, AMC and PD subjects, and the comparisons between kinematic features and groups are summarized in Table 2. On average, subjects of all groups presented similar values in the stride time in both legs. Group subjects showed also similar swing and stance times. PST was around the 65% of the gait cycle in all groups. In relation with bilateral coordination, although on average, DST was similar across groups, patients showed higher values in DST CV than elder and young controls (Figure 1a). GA also found increased in patients and elders with respect to young controls (Figure 1b). Whereas mean values of *φ* were close to the ideal value of 180° in all groups, PD showed increased *φ* deviation than YC but not AMC (Figure 1c). Compared to the YC, PCI values were similar in AMC and significantly higher in the PD group. Statistical significant differences maintained when comparing PD and AMC (Figure 1d).

### 3.2. Kinetics

**Figure 2:**
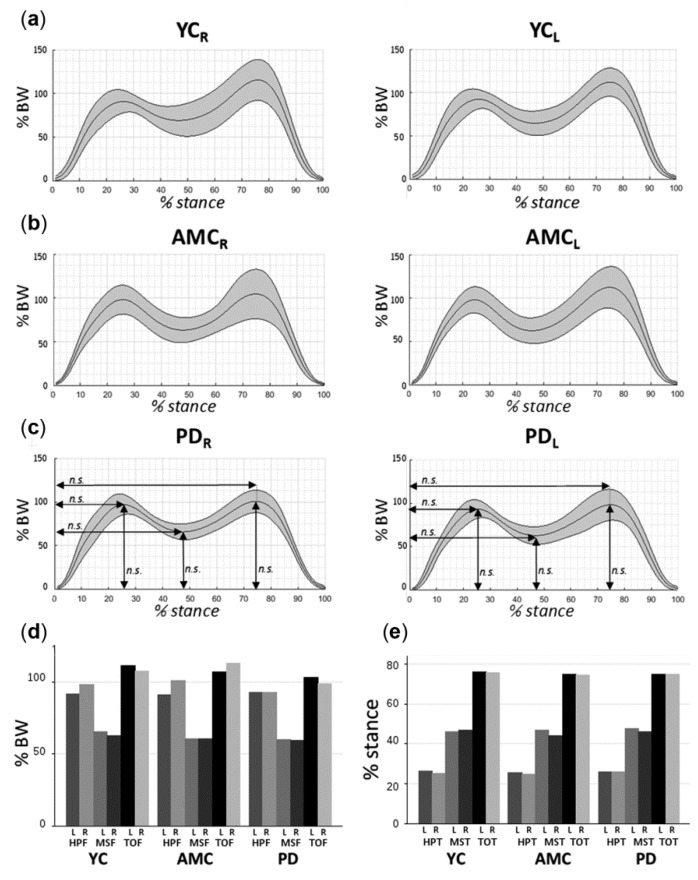
Vertical GRFs profiles for the right and left foot during free-walking in (**a**) YC (n = 17), (**b**) AMC (n = 11), and (**c**) Parkinson’s Disease (PD) (n = 12). (**d**) Evolution of force (**e**) and temporal parameters of the GRFs within the stance cycle across groups. Abbreviations: R: right, L: left, BW: Body weight, n.s.: not significant.

Figure 2a–c displays the average vertical GRFs of YC, AMC and PD subjects during the stance cycle and Table 3 summarizes the kinetic features in each group. 

Similar peak forces were observed between groups at heel-peak, mid-stance and toe-off for right and left foot (Figure 2d). All three groups showed also similar HPT, MST and TOT, which succeeded around the 26%, 46% and 74% of the stance cycle for both limbs, respectively (Figure 2e). On the other hand, variability measures of force and time were found similar across left/right feet and groups, force variability measures were lower at heel-peak than for mid-stance and toe-off. Time variability measures were lower at toe-off than at heel-peak and mid-stance in all groups.

### 3.3. Feature Selection 

Table 4 shows the features that explained DST CV, GA, *φ* deviation and PCI in YC, AMC and PD subjects. Height was found to predict DST CV in all groups, whereas DST predicted DST CV only in control subjects. Age was the better predictor of DST CV in YC. Interestingly, clinical variables such as MMSE, UPDRS, and LEDD ranked with the highest loadings on DST CV in PD subjects, together with swing time and disease duration. For GA, sex was found its better predictor in all groups, whereas weight and height ranked only in controls and did not in PD patients. Elderly groups shown age, MSF, swing time as mutual predictors of GA. Again, GA was forecasted by clinical –status related variables in PD patients (i.e., AFF, disease duration, MMSE and UPDRS), together with the stance, swing and stride times of the right foot. *φ* deviation did not show mutual predictors across groups; thought MSF, swing time and stride time predicted *φ* deviation in the elders. Swing time was found to predict PCI in all groups, whereas DST loaded only in control populations. In the elder groups, sex, MSF, stride time and weight were found to predict PCI. Interestingly, two different prediction patterns (i.e., groups of predictors) were found on the response variables between young and elder populations: in YC, DST, HPF, HPF CV, TOT CV and the number of steps; and in AMC and PD; MSF, swing and stride times. Cadence was found to predict all the response variables in AMC.

### 3.4. Classification Performance

**Figure 3:**
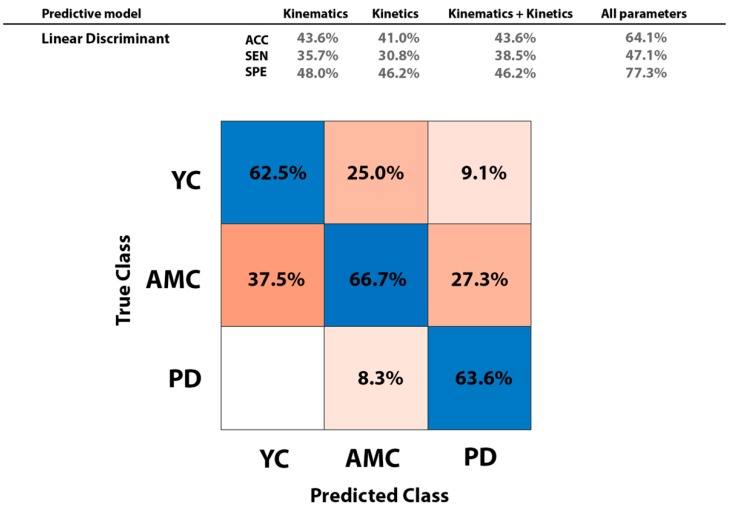
Confusion Matrix of the gait classifier when the 56 predictors (i.e., 4 demographics, 28 kinematics, and 24 kinetics) were included in the model (i.e., classification method: Linear Discriminant, Covariance structure: full).

Figure 3, top (right column) displays results on classification performance when all parameters were included as predictors in the trained model. Performance metrics (i.e., accuracy: ACC; sensitivity: SEN, and specificity: SPE) were slightly higher when kinematic parameters were used as predictors that when kinetic parameters were employed. Classification performance did not increase when kinematic and kinetic parameters were combined and included as regressors in the classifier. Overall accuracy, sensitivity, and specificity in group classification resulted on 64.1%, 47.1%, and 77.3%, respectively. The LDA model classified successfully the 62.5% of YC, 66.7% of AMC, and 66.3% of PD subjects (Figure 3, bottom). 

## 4. Discussion

In this work, we have quantified, compared, and classified the kinematics and kinetics related to free-walking in mildly affected ‘on’ state PD patients and two control groups, corresponding to age-matched controls and young adults. Differences between patients and controls on free-walking were found in kinematic parameters, which are specifically associated with bilateral coordination. The novelty of the study was that all free-walking data were recorded from embedded insole sensors. Specifically, patients presented increased variability in double stance time, higher asymmetry, phase deviation, and lower stability in phase generation. Feature selection analyses on the differential parameters in PD patients explained prediction patterns across groups, showing a key effect in patients’ parameters related to their clinical status. Automatic classification of YC, age-matched (AMC), and patients yielded slightly more accurate data when kinematic predictors were included in the machine learning models than when kinetic predictors were added. The overall accuracy, sensitivity, and specificity of the LDA classifier to distinguish between YC, AMC, and PD patients from the whole set of predictors was 64.1%, 47.1%, and 77.3%, respectively. 

First, Parkinson’s disease patients showed increased variability at double support phase in comparison with AMC and YC (Figure 1a). This result indicates that our sample of patients was less stable in double support phase compared to both control groups. Results are in line with previous studies on gait, which observed increased variability in the double stance time in patients both de novo and in advanced stages of the disease compared to controls [1,2,3,4]. Regarding the higher variability in the double support phase, works using electromyography have associated the difficulties for combining single steps into an accurate and coordinated sequence of movement to disturbed EMG activity patterns in the leg muscle extensors of PD patients [9,11,35]. Dietz and Colombo suggested that the decline in sensitivity of the extensor load receptor function, which has a key contribution to the impaired gait in the elderly, is more pronounced in parkinsonian patients [9]. Importantly, gastrocnemius activity has been demonstrated to induce forward acceleration at late stance [36]. Moreover, our sample of patients showed increased asymmetry than YC, as a result of higher swing difference in duration times between the feet (Figure 1b). In a previous study, Plotnik and colleagues found significant differences in gait asymmetry between young, elders and idiopathic PD patients [37]. Although symmetrical lower limbs performance during gait has often been assumed, asymmetry seems to reflect a natural functional difference between the limbs [38] and thus related to the contribution of each limb to propulsion and control tasks. Nevertheless, several works have reported increased asymmetry in patients with Parkinson’s disease [14,35,39]. In our group of patients, we came across two limitations in order to study gait asymmetry more in depth: one due to the natural history of PD: the asymmetrical clinical onset; and the limited size of the population sample, which makes it difficult to generalize the results. Considering the different asymmetric clinical onset of PD, we performed an exploratory analysis on gait parameters (i.e., swing and stance time ratios) depending on the affected brain hemi-body of PD patients (see Appendix B and Supplementary Figure S1). In spite of the limited sample size of our PD patients’ group, we observed a tendency to a higher stance-ratio in left-PD patients when having into account for the clinical onset of the disease. Thus, one challenge in PD is to ensure in which clinical phase patients are being studied and whether the population is homogeneous related to disease evolution status, since the natural history of this neurodegenerative disease is an asymmetrical onset affecting predominantly one hemisphere and through the evolution of the dopaminergic degeneration affects both hemispheres and is manifested clinically in greater symmetry [40]. 

Parkinson’s disease patients also performed larger deviations from the ideal phase value and exhibited increased phase coordination index compared to both control groups (Figure 1c). Therefore, stability in phase generation seems to be lower in patients than in healthy population; this is an effect prominent on the phase generation index, a measure that represents variability and inaccuracy in phase generation. In patients, phase coordination values were higher as compared to the AMC and much higher as compared to the YC (Figure 1d). This finding is in line with observed alterations on bilateral coordination during gait both in patients de novo [2], mild [39] and with advanced PD [14]. On the other hand, it has been showed that parkinsonian medication improves baseline gait parameters and thus bilateral coordination [37]. The sensitivity of levodopa medication to PD gait kinematic parameters has been tested, finding that swing velocity, peak velocity together with stride length modified with medication, while temporal parameters such as stride and swing duration, stride duration variability were dopaminergic therapy resistant [4]. Parameters related to free-walking were almost similar for YC and AMC, with exception of gait asymmetry. Such similarities in the gait pattern are possibly due because our AMC is younger (i.e., 56.5 years) than population in literature’s studies. The general effects of aging on gait are attributed to age-associated decline in musculoskeletal function [41] as well as changes in the central nervous system [42]. The most apparent change is reduction in gait speed at ages above 65 years of age [43], thought other changes, such as shorter step and stride lengths and decreased ankle extension have also been reported [44]. 

We used the ReliefF algorithm for feature selection due to its potential to capture feature dependencies and also its robustness to over-fitting, being especially useful when strong interactions are expected between features. Feature selection analyses performed over the significant parameters characterizing PD patients (Table 4) showed differences in the parameters that predicted the response variables in each group but importantly, those analyses demonstrated an effect of the clinical status in PD patients. First, greater influence of mid-stance force was observed in elder subjects for all the significant parameters. Considering the key function of mid-stance phase in dynamic balance, a greater influence of mid-stance force could be related to the adaptation by the elderly toward a safer and more stable gait pattern [45]. Foot GRF data during braking and propulsive phases have shown effects of aging [46,47]. Moreover, feature selection analyses confirmed that the clinical status (i.e., MMSE, UPDRS, LEDD, disease duration) affects the variability in double support phase in patients, and that the affected side related to the clinical onset, cognitive status and motor scale predict gait asymmetry. In addition, the pattern of coordination in PD was found to be determined by disease duration and weight. 

In this study, we obtained gait features datasets (i.e., kinematics and kinetics) only from one source: plantar pressure data. There are already many other studies comparing different kinetics and kinematics parameters of gait in Parkinson’s patients. What makes this paper unique is the possibility of embedding such sensors and choose among the parameters. In relation with the moderate performance achieved in automatic classification with LDA, we have to consider that multiclass classification was currently performed over three groups and also the specific characteristics of the groups: (1) Parkinson’s disease patients in our study and accordingly elder controls were younger than the sample groups reported in the literature, (2) The Parkinson’s disease patients population was homogeneous related to disease duration and were explored in the ‘on’ medication phase, that is in their best clinical situation in response to levodopa therapy. Therefore, their gait would improve considerably compared with “off” medication status, acquiring features similar to healthy gait, and (3) the experiment evaluated the specific source assessment (i.e., kinematics, kinetics) that allowed the pressure sensors recordings the higher classification performance not the specific investigation of the high classification performance. The direct comparison of our data with the contributions of Parkinsonian gait studies is difficult because of the low clinical homogeneity of the populations, both in evolution of disease and clinical status related to levodopa therapy response. The rationale of simple data fusion in the datasets employed was that one of our aims was to evaluate which dataset (i.e., kinematics or kinetics) was a better predictor. Thus, our focus was not models performance or high classification accuracy in the combined datasets analyses. Considering that unlike to kinetics, the kinematic parameters included coordination-related features, and that gait disabilities are manifested in PD through a coordination deficit, it seems rationale that kinematic predictors result on a slightly better classification performance than kinetics (Figure 3, top). Overall classification performance of the LDA machine learning model (Figure 3, bottom) yielded a 64.1% of accuracy, 47.1% of sensitivity and 77.3% of specificity. 

In summary, in-shoe insoles with high resolution allow for examination of kinematic and kinetic parameters related to locomotion in Parkinson’s disease using free-walking at an individual pace. In the first stages of the disease and under Levodopa therapy, the patient population presented increased variability in double support time, higher asymmetry, and lower stability in phase generation compared to controls. We show that in-shoe measurement system recordings are useful to examine the differences in kinematics and kinetics referred to gait patterns across populations, which could be especially interesting to determine longitudinal changes due to disease evolution and interventions such as new drugs or rehabilitation programs in gait-disabled populations. Finally, this experiment was a pilot study and we aim to perform studies over larger population sizes and other gait disturbances in future work.

## 5. Conclusions

In-shoe pressure measurement systems offer a great potential to examine disabilities related with gait. Free-walking at a natural pace demonstrates that Parkinson’s patients in a mild stage of the disease during the ON clinical status show significant alterations in bilateral coordination. 

## Figures and Tables

**Table 1:** Demographic parameters (in the form of median ± standard deviation values) and comparisons across groups (χ^2^_kw_ and *p*-values are reported).

	YC	AMC	PD	χ^2^_Kruskal Wallis_	*p*-Value
Age [years]	29.5 ± 3.63	56.5 ± 12.4 *	57 ± 7.84	27.9	<0.001
Weight [kg]	68 ± 9.28	70 ± 11.03	81 ± 12.92	5.05	0.08
Height [m]	1.68 ± 0.08	1.70 ± 0.07	1.75 ± 0.09	1.35	0.51
BMI [kg/m^2^]	23.30 ± 2.64	24.72 ± 2.07	26.47 ± 4.48	5.73	0.06

YC = Young control subjects, AMC = age-matched control subjects, PD = Parkinson’s disease patients.* Significant differences between YC and AMC.

**Table 2:** Kinematic parameters (n = 28) related with free-walking (in the form of median ± standard deviation values) and comparison across groups (χ^2^_kw_ and *p*-values are reported).

	YC	AMC	PD	χ^2^_KW_	*p*-Value
Nr steps	524.5 ± 52.2	524.5 ± 32.7	553 ± 31.3	2.42	0.30
Distance (m)	377.3 ± 62.5	384.8 ± 57.5	381 ± 46.2	0.68	0.71
Step length [m]	0.71 ± 0.07	0.73 ± 0.07	0.70 ± 0.06	1.41	0.50
Speed [m/s]	1.26 ± 0.21	1.27 ± 0.21	1.27 ± 0.18	0.14	0.93
Cadence [strides/s]	1.75 ± 0.17	1.73 ± 0.12	1.83 ± 0.13	3.46	0.18
R stride time [s]	1.13 ± 0.11	1.14 ± 0.06	1.10 ± 0.08	1.16	0.56
L stride time [s]	1.12 ± 0.11	1.13 ± 0.06	1.10 ± 0.09	1.41	0.49
R stance time [s]	0.76 ± 0.08	0.73 ± 0.04	0.69 ± 0.05	2.94	0.23
L stance time [s]	0.75 ± 0.08	0.73 ± 0.05	0.71 ± 0.05	2.79	0.25
R swing time [s]	0.38 ± 0.03	0.38 ± 0.03	0.37 ± 0.03	2.01	0.37
L swing time [s]	0.38 ± 0.03	0.38 ± 0.03	0.37 ± 0.03	2.20	0.33
R stride time CV	2.37 ± 1.29	2.58 ± 0.35	2.41 ± 1.04	0.08	0.96
L stride time CV	2.42 ± 1.40	2.22 ± 0.39	2.14 ± 1.36	1.82	0.40
R stance time CV	1.25 ± 5.25	1.36 ± 1.87	1.72 ± 2.20	1.74	0.42
L stance time CV	1.51 ± 5.11	1.42 ± 2.5	2.05 ± 11.29	2.47	0.29
R swing time CV	3.38 ± 0.70	3.03 ± 0.35	3.32 ± 0.52	3.93	0.14
L swing time CV	3.14 ± 0.69	2.92 ± 0.44	3.14 ± 0.53	3.02	0.22
R PST (%)	65.09 ± 1.62	65.19 ± 2	64.72 ± 1.77	0.51	0.77
L PST (%)	65.34 ± 1.48	64.89 ± 2.07	65.00 ± 1.94	1.54	0.46
R PSWT (%)	33.98 ± 1.48	34.23 ± 2.03	34.66 ± 1.49	1.65	0.44
L PSWT (%)	33.50 ± 1.31	34.50 ± 2.01	33.78 ± 1.73	3.69	0.16
DST [s]	0.18 ± 0.03	0.17 ± 0.02	0.17 ± 0.02	2.79	0.25
DST CV	5.06 ± 1.56	5.63 ± 1.11	8.45 ± 4.34 (0.01) ^+^	14.70	0.001
φ [degrees]	180.10 ± 1.64	181.31 ± 2.78	181.55 ± 5.58	1.22	0.54
φ CV	1.35 ± 0.28	1.30 ± 0.32	1.51 ± 0.46	5	0.08
φ deviation	1.46 ± 0.32	1.55 ± 0.63	2.58 ± 1.29 (0.003)	10.73	0.005
GA	0.91 ± 0.56	2.17±1.06 (0.01)	2.77 ± 2.50 (0.013)	11.98	0.003
PCI	2.82 ± 0.54	2.92 ± 0.83	4.36 ± 1.50 (0.003) ^+^	12.15	0.002

Numbers in the parentheses represent the post-hoc *p*-values compared to YC (i.e., when significant differences across groups exist). ^+^ Statistical significance between PD and AMC.

**Table 3:** Kinetic parameters (n = 24) related with free-walking (in the form of median ± standard deviation values) and comparison across groups (χ^2^_kw_ and *p*-values are reported).

	YC	AMC	PD	χ^2^_Kruskal Wallis_	*p*-Value
R HPF [% BW]	0.92 ± 0.13	0.94 ± 0.17	0.93 ± 0.14	1.13	0.57
L HPF [% BW]	0.98 ± 0.12	1.00 ± 0.16	0.93 ± 0.12	0.62	0.73
R MSF [% BW]	0.66 ± 0.16	0.60 ± 0.12	0.60 ± 0.09	1.15	0.56
L MSF [% BW]	0.62 ± 0.14	0.60 ± 0.14	0.60 ± 0.10	1.05	0.59
R TOF [% BW]	1.12 ± 0.25	1.04 ± 0.23	1.03 ± 0.16	1.71	0.43
L TOF [% BW]	1.08 ± 0.17	1.10 ± 0.27	0.99 ± 0.19	2.14	0.34
R HPF CV	5.20 ± 1.02	4.75 ± 1.88	6.08 ± 1.63	3.07	0.22
L HPF CV	4.68 ± 1.93	4.30 ± 2.08	6.41 ± 2.07	2.97	0.23
R MSF CV	6.80 ± 3.98	8.47 ± 6.06	7.25 ± 5.17	1.65	0.44
L MSF CV	6.75 ± 4.33	9.16 ± 4.30	7.17 ± 5.05	0.46	0.80
R TOF CV	7.78 ± 3.13	6.88 ± 8.07	7.27 ± 3.39	0.10	0.95
L TOF CV	5.94 ± 5.36	7.24 ± 4.72	6.15 ± 3.99	1.19	0.55
R HPT [% stance]	26.43 ± 3.56	25.80 ± 1.68	25.88 ± 1.93	0.37	0.83
L HPT [% stance]	25.33 ± 3.22	25.76 ± 2.16	25.86 ± 1.84	1.63	0.44
R MST [% stance]	46.07 ± 4.54	46.98 ± 5.82	47.59 ± 2.58	0.52	0.77
L MST [% stance]	46.82 ± 2.74	44.19 ± 4.01	46.25 ± 4.92	3.57	0.17
R TOT [% stance]	75.87 ± 2.25	74.80 ± 1.92	74.73 ± 2.33	3.77	0.15
L TOT [% stance]	75.62 ± 1.69	74.22 ± 3.75	74.72 ± 1.60	2.58	0.27
R HPT CV	4.65 ± 4.05	3.56 ± 1.93	4.59 ± 1.76	4.51	0.10
L HPT CV	5.04 ± 1.76	3.52 ± 2.55	4.34 ± 2.66	0.70	0.70
R MST CV	6.18 ± 4.31	3.18 ± 3.33	4.46 ± 2.02	4.91	0.09
L MST CV	3.83 ± 2.18	3.96 ± 2.51	3.63 ± 2.02	0.01	0.99
R TOT CV	1.21 ± 0.83	1.70 ± 1.01	1.39 ± 0.68	3.89	0.14
L TOT CV	1.33 ± 0.78	1.54 ± 1.71	1.37 ± 0.38	1.62	0.44

**Table 4:** Feature selection analyses of the gait parameters that highlighted in PD patients. The most important predictors are reported for each parameter and group.

**DST CV**	***YC***	**Weight**	***AMC***	**Weight**	***PD***	**Weight**
	Age	0.055	DST	0.036	MMSE	0.075
	R TOT CV	0.019	L stride time	0.026	UPDRS	0.046
	DST	0.016	Cadence	0.013	LEDD	0.026
	Height	0.010	Distance	0.006	Height	0.022
	R HPF CV	0.010	Weight	0.003	R swing time	0.021
	L HPF	0.009	Height	0.002	Disease duration	0.005
	Nr steps	0.007				
**GA**	***YC***	**Weight**	***AMC***	**Weight**	***PD***	**Weight**
	Sex	0.133	Sex	0.067	Sex	0.145
	DST	0.015	Age	0.028	AFF	0.065
	R TOT CV	0.012	Weight	0.028	Disease duration	0.024
	L HPF	0.011	L MSF	0.019	Age	0.024
	Weight	0.010	L swing time	0.016	R MSF	0.022
	Height	0.008	R swing time	0.014	MMSE	0.017
	Distance	0.005	Height	0.003	UPDRS	0.011
	Nr steps	0.004	Cadence	0.001	R stance time	0.010
	R HPF CV	0.004			R swing time	0.008
					R stride time	0.001
***φ* dev**	***YC***	**Weight**	***AMC***	**Weight**	***PD***	**Weight**
	L HPF	0.016	L MSF	0.020	Sex	0.214
	R TOT CV	0.016	L swing time	0.016	R stride time	0.029
	Nr steps	0.010	L stride time	0.010	Age	0.022
	DST	0.009	Cadence	0.006	R swing time	0.021
	R HPF CV	0.002			R MSF	0.009
					Height	0.006
					Disease duration	0.004
					Weight	0.001
**PCI**	***YC***	**Weight**	***AMC***	**Weight**	***PD***	**Weight**
	DST	0.019	Sex	0.027	Sex	0.227
	L HPF	0.015	L MSF	0.023	Weight	0.042
	R swing time	0.010	L stride time	0.019	R stride time	0.028
	R TOT CV	0.008	L swing time	0.014	R MSF	0.024
	Nr steps	0.008	Weight	0.006	R swing time	0.020
	R HPF CV	0.002	DST	0.006	Disease duration	0.020
			Cadence	0.004		

## Supplementary Materials

The following are available online at http://www.mdpi.com/1424-8220/18/12/4224/s1, Supplementary Table S1: Overall accuracy (ACC), sensitivity (SEN) and specificity (SPE) in gait detection of the predictive models with different predictors’ datasets while validated in a leave-one out scheme, Supplementary Figure S1: Boxplots of the swing time ratio and stance time ratio (normalized to the right foot) across stepping movements and groups while considering disease lateralization in PD patients.

## Appendix A

Automatic classification was performed with the Classification Learner Toolbox on the four datasets while evaluating ten classification methods: coarse tree, linear support vector machine (SVM), Quadratic SVM, Cubic SVM, Fine K-nearest neighbor (KNN), Medium KNN, Cubic KNN, Cosine KNN, and Linear Discriminant. Supplementary Table S1 detail the classification performance of the generated models, based on the following metrics:

$$Accuracy=100\times \frac{TP+TN}{TP+FP+TN+FN}$$

$$Sensitivity=100\times \frac{TP}{TP+FN}$$

$$Specificity=100\times \frac{TN}{TN+FP}$$

where *TP*, *TN*, *FP*, and *FN* are the number of true positives, true negatives, false positives and false negatives, respectively. In most predictive models, analysis including kinematic data as predictors resulted on a greater classification performance. In fact, the inclusion of dynamic data did not increase accuracy, except in Coarse Tree and Quadratic SVM models. Demographic data (i.e., sex, weight, height and BMI) increased overall accuracy, sensitivity and specificity, especially in the linear discriminant model.

## Appendix B

Kruskal-Wallis tests were employed to test for differences on gait asymmetry depending on the affected hemi-brain (i.e., swing/stance time ratios). The Kruskal-Wallis test comparing the mean swing time ratio between groups did not report significance (*X*^2^ = 7.29, *p* = 0.06), but the Kruskal-Wallis test for the stance time ratio resulted in significance (*X*^2^ = 8.87, *p* = 0.03), meaning that on average, the stance time ratio was significantly different among groups. Despite post-hoc tests did not surpass the significance threshold in any group comparison, PD patients affected in the left-side (n = 4) tended to differentiate in the stance time ratio in respect with the other groups.
