# Supplementary material for: Kinematic and Kinetic Patterns Related to Free-Walking in Parkinson’s Disease
Source: Sensors (Basel). 2018 Dec 1;18(12):4224. doi: 10.3390/s18124224 (PMC6308417; doi:10.3390/s18124224)
Supplement: Supplementary file 1 [file sensors-18-04224-s001.pdf]

Supplementary Table S1. Overall accuracy (ACC), sensitivity (SEN) and specificity (SPE) in gait detection of the predictive models with different predictors' datasets while validated in a leave-one out scheme. Characteristics of the classifiers: Coarse tree- Split Criterion = 'gdi', MaxNumSplits = 4; Linear SVM- Kernel function = Linear, Kernel scale = automatic, Box constraint level = 1, Multiclass method = one-vs-one, Standardize data = true; Quadratic SVM- Kernel function = Quadratic, Kernel scale = automatic, Box constraint level = 1, Multiclass method = one-vs-one, Standardize data = true; Cubic SVM- Kernel function = Cubic, Kernel scale = automatic, Box constraint level = 1, Multiclass method = one-vs-one, Standardize data = true; Fine KNN- Number of neighbors = 1, Distance metric = Euclidean, Distance weight = true ; Medium KNN- Number of neighbors = 10, Distance metric = Euclidean, Distance weight = true; Cubic KNN- Number of neighbors = 10, Distance metric = Minkowski, Distance weight = true; Cosine KNN- Number of neighbors = 10, Distance metric = Cosine, Distance weight = true; Linear Discriminant: Covariance structure: full; A 39-fold (i.e., leave-one out) cross validation scheme was employed for all the analyses.

**Predictive model**		**Kinematics**	**Kinetics**	**Kinematics + Kinetics**	**All parameters**
Coarse Tree	*ACC*	46.2	25.6	48.7	43.6
	SEN	36.4	30.8	44.4	37.5
	SPE	50.0	23.1	50.0	45.2
Linear SVM	*ACC*	61.6	38.5	51.3	53.9
	SEN	50.0	30.0	33.3	36.4
	SPE	65.6	41.4	59.3	60.7
Quadratic SVM	*ACC*	48.7	28.2	53.9	48.7
	SEN	31.2	26.7	36.4	25.0
	SPE	60.9	29.2	60.7	59.3
Cubic SVM	*ACC*	53.9	30.8	48.7	48.7
	SEN	36.4	25.0	30.8	30.8
	SPE	60.7	33.3	57.7	57.7
Fine KNN	*ACC*	43.6	35.9	38.5	43.6
	SEN	23.1	35.7	30.0	27.3
	SPE	53.9	36.0	41.4	50.0
Medium KNN	*ACC*	53.9	56.4	51.3	51.3
	SEN	50.0	37.5	36.4	33.3
	SPE	56.5	61.3	57.1	62.5
Cubic KNN	*ACC*	53.9	56.4	51.3	43.6
	SEN	43.8	45.5	28.6	18.2
	SPE	60.9	60.7	56.3	53.6
Cosine KNN	ACC	51.3	51.3	51.3	59.0
	SEN	37.5	55.6	42.9	45.5
	SPE	54.8	50.0	53.1	64.3
Linear Discriminant	*ACC*	43.6	41.0	43.6	64.1
	SEN	35.7	30.8	38.5	47.1
	SPE	48.0	46.2	46.2	77.3

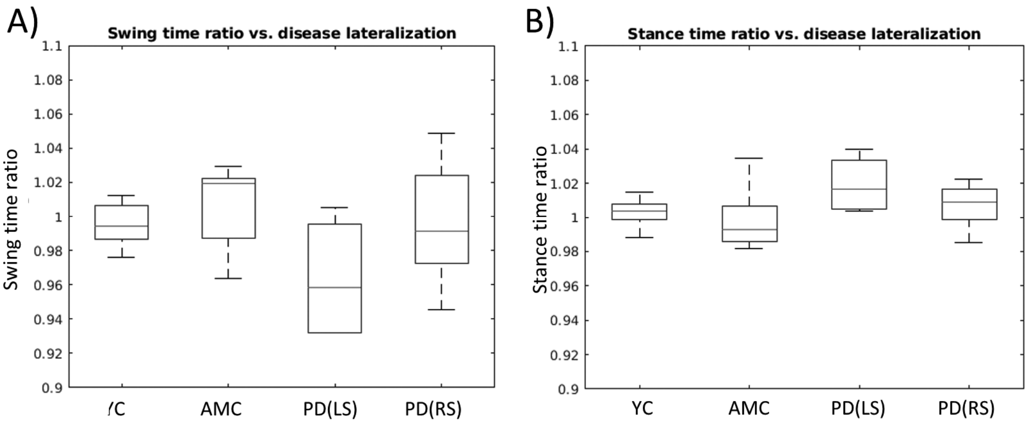

Supplementary Figure S1. Boxplots of the (**A**) swing time ratio and (**B**) stance time ratio (normalized to the right foot) across stepping movements and groups while considering disease lateralization in PD patients. Abbreviations: RS = right side. LS = left side.
